# “And Then He Got into the Wrong Group”: A Qualitative Study Exploring the Effects of Randomization in Recruitment to a Randomized Controlled Trial

**DOI:** 10.3390/ijerph17061886

**Published:** 2020-03-14

**Authors:** Birthe Andrea Lehmann, Lara Lindert, Silke Ohlmeier, Lara Schlomann, Holger Pfaff, Kyung-Eun Choi

**Affiliations:** 1 Communication Science, University of Amsterdam, 1001 NH Amsterdam, The Netherlands; 2 Institute of Medical Sociology, Health Services Research and Rehabilitation Science, University of Cologne, 50933 Köln, Germany; lara.lindert@uk-koeln.de (L.L.); silke.ohlmeier@uks.eu (S.O.); lara.schlomann@uk-koeln.de (L.S.); holger.pfaff@uk-koeln.de (H.P.); anna.choi@uk-koeln.de (K.-E.C.)

**Keywords:** recruitment barriers, randomization, RCT, qualitative research, case managers

## Abstract

Background: Randomized controlled trials (RCTs) are regarded as the most internally valid means of estimating the effectiveness of complex public health interventions, but the recruitment of participants can be difficult. The aim of this study was to explore factors that may have affected the recruitment of employees with musculoskeletal disorders (MSDs) to a multicenter worksite health promotion program from the perspective of recruiting case managers. Methods: Factors in recruitment to the RCT were explored using three focus group discussions with case managers. Data were processed using MAXQDA and analyzed with a combination of content and sequence analysis. Results: Findings showed that randomization is a major challenge for recruitment. Case managers adapted their communication with, and approaches to possible participants because of the randomization design and employed coping strategies to compensate for allocation into the control arm of the study. Perceptions of the superiority of the intervention group over the control group, perceptions of the (mis)match of participants to one of the groups, as well as the understanding of the necessity of randomization for effectiveness evaluations, further affected recruitment. Perceived expectations of possible participants and their (emotional) reactions to the randomization allocation also complicated recruitment. Conclusion: We were able to gain insight into the challenges of randomization for the recruitment of participants to a multicenter RCT. This study assisted the development of strategies to overcome barriers in the ongoing implementation process of the trial (i.e., the adaption of best practice information sheets and newsletters). There remains a need to develop effective interventions to help those recruiting to trials.

## 1. Introduction

Randomized controlled trials (RCTs) have long been regarded as the gold standard for evaluating the effectiveness and safety of not only clinical studies, but also complex public health interventions [1,2]. They are still regarded as the most internally valid means of estimating the overall effects of interventions, even though it is recognized that RCTs are not always feasible for complex interventions. Multicenter RCTs are often seen as the design of choice to generate the necessary evidence for funders and policymakers to implement interventions that prove to be effective into standard care [2,3,4]. However, the general usefulness of experimental intervention studies has been looked upon with skepticism and the recruitment of participants to RCTs can be very challenging [5]. In fact, many studies report underenrollment [6], with a review finding that less than a third of multicenter RCTs achieve their original recruitment target [7]. It has been found that the communication style of recruiters plays an important role in participant understanding of RCTs and their willingness to join such a study [8]. A systematic review has shown that participants and recruiters often have strong preferences for a treatment group, which can affect recruitment [9] and may lead to poor participant adherence if they are not allocated to their preferred treatment group [6].

In spring 2017, a four-year trial to test the effectiveness of a corporate health management program for employees with musculoskeletal disorders (MSDs) was implemented in Germany. It intends to offer targeted support tailored to the individual needs of sick or vulnerable employees. The program is an approach with which to combat insufficient cross-sector healthcare cooperation for employees with MSDs, by establishing close cooperation and coordination between the relevant health insurance carriers, pension providers, and companies. A central innovation of the program is the implementation of case managers, who act as contact persons for policyholders and who coordinate the interfaces between the various parties. In Germany, many companies have affiliated health insurance funds that support them in corporate health management. The case manager is an employee of the health insurance fund and leads the company employees through the healthcare process and involves them in all planning and decision-making processes. During the trial, they additionally have the task of recruiting employees. Implementing case managers for the recruitment and coordination of such a program has advantages and disadvantages. On the one hand, the case managers play a unique and important role in that they are close to the individual employees, but also because they can oversee the necessary processes involved in coordinating the program. If the trial proves to be effective, the program can be transferred into standard care without further external assistance. On the other hand, as employees of health insurance funds, they have little research experience, which might affect the recruitment process during the trial.

In this study, we explore randomization as a factor in the recruitment of participants to a worksite health intervention from the perspective of recruiting case managers with focus groups and discuss how this can help to better understand the factors affecting recruitment and to inform and improve the ongoing trial. Previous research has demonstrated how qualitative methods can be used to understand factors affecting recruitment to RCTs on both recruiter and participant sides and how this, in turn, can be used to facilitate the workings of such a research strategy [8,10].

## 2. Materials and Methods

### 2.1. Setting

**Figure 1:**
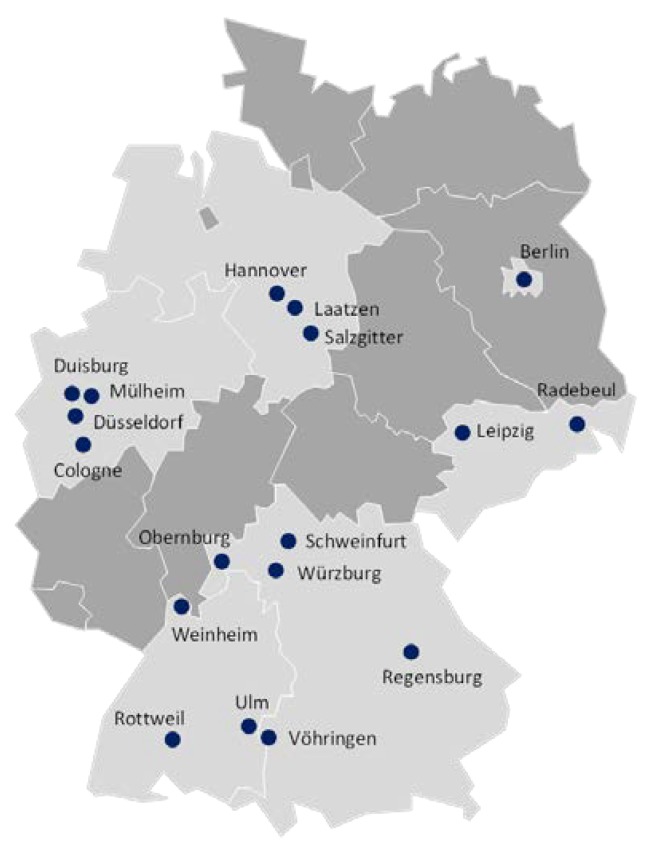
Locations of the participating businesses in 22 cities across Germany.

**Figure 2:**
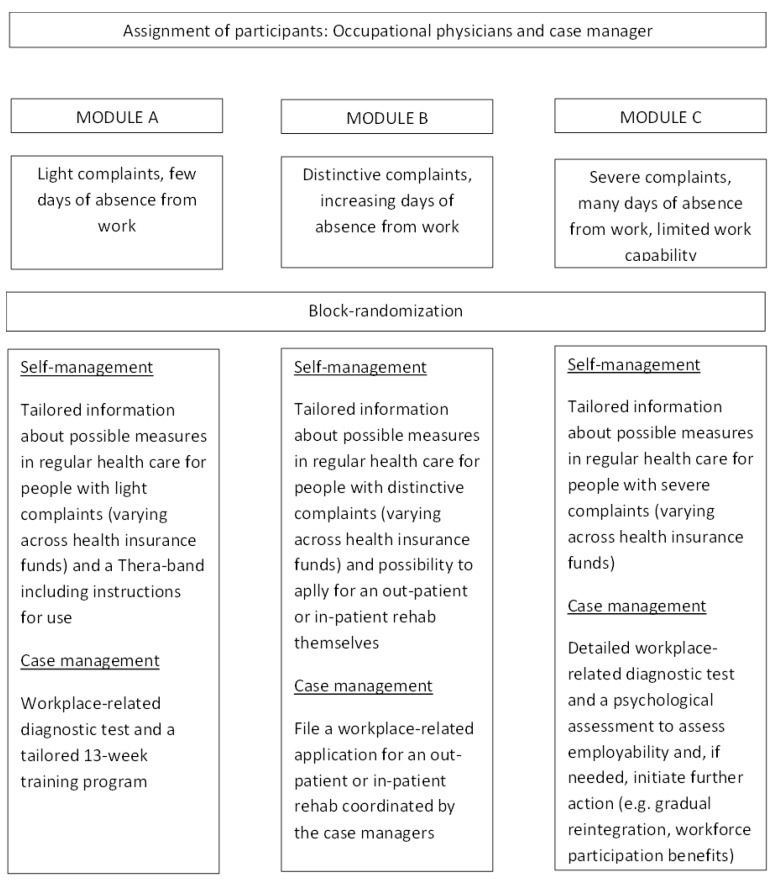
Assignment of employees to musculoskeletal disorders (MSD)-specific modules.

The RCT was implemented in 22 business locations in 20 cities across Germany (see Figure 1). It has a multimodal structure and intends to establish workplace-relevant, individualized, and cross-sector healthcare measures. Before entering the study, employees were allocated to one of three modules. Depending on the stage of the disease and the impact on the employee, these measures may be early intervention (MODULEA), rehabilitation (MODULE B), or reintegration (MODULE C). Afterwards, employees who agreed to participate in the study were randomly assigned to either a self-management group, which represents the standard care currently offered in Germany, or the case management group. In the case management group, employees received more work-related diagnostics and support by a case manager in starting training or rehabilitation. See Figure 2 for a description of the modules. Combined, the business locations comprised approximately 45,000 employees, of whom an estimated three to four thousand might be eligible for participation in the program. Participating businesses included varying professions, including blue collar workers such as mechanics and steel workers, as well as white collar workers such as corporate consultants and accountants. These employees were, in some cases, shift workers, and some had to travel for their work. Business sectors included, amongst others, steel and metal manufacturers, the automotive industry, technology ventures, the trade and service industry, and administration and government agencies.

### 2.2. Participants and Procedure

Fifteen health insurance funds participated in the implementation of the trial, providing 22 case managers, including three substitutes. The number of required case managers was determined with the assumption that case managers will spend 10% to 15% of their working hours on the trial. Only one case manager was specifically employed full time for the trial. Department managers of the health insurance funds assigned case managers to the project. Case managers were customer consultants with training as, amongst other positions, social security employees and medical assistants in occupational-, alternative and psychotherapy. Medical know-how was considered an advantage for the project. Some of the company health insurance funds primarily provided insurance for the employees of one company and were at the same location as the companies, while others provided insurance for the employees of several different companies and were located at a different location than the companies. The recruitment goals of case managers varied, depending on the size of the affiliated business and the number of insured employees in that business. All case managers received extensive two-day training to learn about the study design and the multimodal program before the start of the trial. They received a field manual that explained the necessity of the study design, the specific proceedings of the program, and advice on the organization of the communication with participants. As the trial proceeded, case managers were also supported in recruitment activities, with regular meetings to allow for the exchange of best practices and materials used during recruitment by case managers (i.e., communication strategies, folders with self-management offers, positive employee examples as advocates in company magazines).

All case managers of the 15 health insurance funds included in the trial, were approached by e-mail. Eighteen out of 22 case managers participated in three focus group discussions in February and March 2018, eight months after the start of recruitment. Sampling for the three focus groups was based on the distance of the case managers to three chosen locations across Germany, resulting in a distribution of six case managers in the eastern part of Germany (FG1), four case managers in the western part of Germany (FG2), and eight case managers in the central part of Germany (FG3). Discussions were held in premises provided by different project partners (a rehabilitation center and two health insurances). Case managers were provided with information concerning the purpose of the focus groups, anonymity, and confidentiality conditions, and the voluntariness of participation before each focus group discussion. The group discussions were conducted by one of the authors (KEC), a psychologist with a PhD in medical sciences, who had no dual relationship with the participants. Participants were familiar with the moderator from previous project meetings and knew her goal of conducting the focus groups. A semi-structured topic list was used. In each focus group, one research assistant was present to take notes. The group discussions were audiotaped, lasted for approximately 120 min with a short break in the middle, and were transcribed verbatim. Transcripts were not returned to participants for comments. Informed written consent was acquired. The Ethics Commission of Cologne University’s Faculty of Medicine reviewed and approved the study.

### 2.3. Data Collection and Analysis

Data were collected by means of three semi-structured focus group discussions. Focus groups were chosen as a method to stimulate discussion about topics that might not surface in individual interviews and to allow for an exchange of experiences and applied strategies between case managers [10]. After introducing themselves and describing the status of the project, case managers were asked about facilitating and inhibiting factors in the cooperation with the different parties of the project. The topic list included the topic of randomization, as this had been identified as a possible challenge for the recruitment of employees by the case managers in previous meetings. Data were processed with MAXQDA (VERBI Software, 2019, Berlin, Germany). This exploratory study was analyzed with content and sequence analysis, utilizing a general inductive approach [11,12]. The data were initially approached by two coders (BAL, PhD in Psychology and SO, sociologist) who developed a code system. This code system was then applied by three different coders (KEC, PhD in medical sciences, LL, rehabilitation scientist, and LS, health economist) who each coded one of the focus group discussions. Finally, one of the first coders (BAL) repeated the coding process for all the material to ensure that all coders had the same interpretation of the codes. All authors then discussed and agreed on the interpretation of the data. Following analysis, quotes were selected on the basis of their representativeness of the findings and subsequently translated from German into English. This qualitative study adheres to the consolidated criteria for reporting qualitative studies (COREQ guidelines), developed for explicit and comprehensive reporting of interview and focus group studies [13].

## 3. Results

Focus group discussions were held with 18 of the 22 case managers that are participating in the project (5 out of 6 men and 13 out of 16 women), representing all participating business locations and health insurance funds. Randomization surfaced as a central challenge for the recruitment of employees by case managers. We, therefore, chose to focus on how randomization was reported to affect the recruitment process and the implementation of the program in our analysis. We identified two different topic blocks using sequence analysis of the parts of the discussions that focused on the topic of randomization: 1. Case manager perceptions of the effect of the randomization design on themselves, and 2. Their perceived effect of the design on participants. These blocks were further divided into sub-questions. For the perceived effect on themselves: (1) How do they deal with the randomization design? (2) How do they perceive randomization and allocation into the groups? (3) How does that make them feel? (4) What is the perceived impact on the company health insurance funds and the study? Sub-questions for the perceived effect on participants were: (5) How do they perceive randomization and the allocation into the groups? (6) How do they react and what are the consequences for the study? Within the sub-questions, we identified distinctive topics that answered these questions, which will be described in the following paragraphs.

### 3.1. Effect on Case Managers

#### 3.1.1. How Do They Deal with the Randomization Design?

##### Communication with Participants

Some case managers reported positive encounters with participants, when they explained the necessity of the study design.

[…] On the basis of the meetings, I contact the insured person, inform them, and tell them from the beginning, it will go like this and that and that, and there are two comparison groups. And so far, I personally haven’t had any negative experiences. […]

(CM11, FG2)

The majority of case managers reported that communication with possible participants was difficult due to the randomization procedure and having to explain the two different groups: Self-management vs. case management. They repeatedly reported that they had to explain to participants that the randomization procedure could not be affected by the case managers, and that it was based on chance.

Precisely. It is harder to convey this to them (participants who expect to be allocated to the treatment group) in this study and that I don’t really have any influence on it. That’s the very problem.

(CM16, FG3)

Communication was described as effortful and time-consuming due to the RCT-design of the study, often involving long conversations and having to schedule several meetings, in some cases, across several weeks before being able to recruit a participant to the study.

It takes a long time, the whole conversation is more difficult, effortful and considerably longer, because you totally have to fight for this self-management and in the end, the client doesn’t lose anything, when they participate. On the contrary. In any case, it is very, very difficult to start, when you do not start at zero, but somewhere completely different, where someone wants to get this case management at all costs. […]

(CM12, FG2)

Case managers additionally said that they still lacked routine in communicating about the study at this stage of the recruitment process. What made communication about the study particularly challenging, according to case managers, was that many potential participants would come into the first meeting with certain expectations that were often caused by conversations with the occupational physician, their employer, or something they had read on a poster promoting the project. This led to the perception that case managers often needed to manage the expectations of participants. Many case managers, therefore, preferred participants who would come into the first meeting without any prior information about the specifics of the study: People they had approached first.

You also have, if I may say something about this briefly, different expectations. Whether the insured person has read a poster or whether they come to us directly and only get into the self-supporting group. Then the disappointment will be greater than when I approach them at the counter and explain the project. […]

(CM16, FG3)

Other factors that affected communication about the study design, according to case managers, involved the occupation of the participants (i.e., white collar workers wanting more information about the study than blue collar workers), how well they knew the participant personally (being more difficult when they knew them well), and which module the participant would fit into (Module A being perceived as most difficult to explain).

##### Strategies to Cope with Randomization

Case managers employed different strategies to communicate with possible participants, ranging from talking about the study as neutrally as possible, through saying that both groups would receive better treatment than they would receive in standard care, to emphasizing that participation in the trial was a privilege. One communication strategy was to emphasize the individual problem of the insured person, instead of starting off by introducing the study.

The other one is to give advice much more neutrally and they will be satisfied with their solution, because it’s about them. They then know, okay, the employee from the health insurance fund cares about me and found a solution for me, or at least has a point of departure to do something about their problem. If and how it helps, that’s another story. […]

(CM16, FG3)

Several case managers reported that they coped with participants being allocated into the self-management arm of the study by offering compensation with alternative training programs available to them.

When the HR advisors send us people, they tell them they have something, go to the health insurance. And when they then come back, ‘they didn’t have anything’. Difficult. We now try to compensate for that. What does compensate mean? We offer different interventions, so that there is something to it and slowly people are coming.

(CM15, FG3)

This was especially the case for allocation to the self-management group in Module A. Some case managers had prepared a folder including available alternatives for participants who would be allocated to the self-management group, so that the participants would have more to go home with than the Thera-band.

In our case it’s like that, we actually got that from you (CM7), with this folder and we primarily have it for the self-management group, a folder with the logo on the front and we put in it all the ideas from health insurance fund X, price reductions in the gym, what we can offer, all the prevention courses, rehabilitation sport. And then we give them the folder, the Thera-band, the information leaflet and the questionnaire and then they don’t just leave the room with a Thera-band.

(CM13, FG3)

Some case managers questioned whether offering these alternatives in the self-management training would disturb the comparability of the two groups for the study.

But then the group comparison is not worth anything anymore. When you take the control group, which has the self-management and give them something else anyway, then you cannot compare them anymore.

(CM13, FG3)

In some instances, case managers reported not offering participation in the trial. This was said to be done when participants would not benefit from being allocated into the self-management group, as was perceived to be the case for employees in Module C.

Because we had the problem, if they got into the self-management group we said: Don’t randomize.

(CM5, FG1)

#### 3.1.2. How Do Case Managers Perceive Randomization?

##### Comprehension Study Design

In general, case managers seemed to be understanding the necessity of randomization for the trial.

In our case, or in my case, when I conduct the conversations, I couldn’t say that they are really disappointed, because I try beforehand to point out that we need both groups to get a result in the end. […]

(CM18, FG3)

Some case managers reported that they perceived an order or pattern in the randomization procedure, so that they would know beforehand which group a participant would be allocated to.

And then the fact that I don’t have an influence over the randomization and that the result is always a surprise. However, I realized that there is a pattern. That means, when I know the last person got into the case management group, then I know that the next person will get into the self-management group. This is the pattern that I have seen in my case.

(CM4, FG1)

##### Group Perception

In general, case managers reported being happy to be involved in the trial, because it was seen as beneficial to be able to provide participants with novel treatments.

In general, everyone at Company X thinks the project is great and we would be really happy if it would really start soon (inclusion of participants), so that we can also do some good for the insured people, of course. And we’ll see what the future brings.

(CM11, FG2)

Group perceptions seemed to be generally dependent on whether case managers perceived a (mis)match of participants and their allocated group. Perceptions of a good group match were reported by case managers for people who were allocated to the group that they preferred to begin with, and, in general, for participants who had not been active before and who experienced an improvement of their health status after participating.

Many of those who I had in this project are employees who have problems but hadn’t done anything about them yet. For them, it was a good starting point and they are partly done with the program and want to continue to do something for their health. The initial test also already had some positive feedback, especially in pain reduction and then the indication to do something about one’s health.

(CM15, FG3)

Case managers reported that participants were often chosen to be approached for the study because it was known that they had physical complaints that would fit with the program or that they had a certain number of sick leave days.

A group mismatch of participants was perceived for several reasons. It was reported that younger participants would generally prefer training with fitness equipment, instead of going into inpatient rehab, while middle-aged, physically hard-working men were not seen as a good match for self-management training.

I don’t know, it might also be a bit dependent on the clientele. In our case, they are almost all men. It’s a factory, where it’s hands-on, a lot of physical work. Accordingly, they are (blue collar) workers, who are comparably robust, some may be already a bit overweight. Basically, it’s the working class. They are around 40, 50 years of age, and some have back problems. […] When you have someone like this standing in front of you, comparably robust and so on, a Thera-band isn’t really hip/fancy. Then they are already looking at me funny. And it’s simply, a man will also be less likely do a yoga course or something and it’s the same with the Thera-band. It somehow doesn’t fit with the clientele. It’s the same with rehab. When I have a young person, they would rather train on fitness equipment than lie on the mat and do exercises. That’s how it is. There are exceptions, but usually, it’s like that.

(CM10, FG2)

Some of the potential participants had professions that made it difficult for them to incorporate 13 weeks of training into their daily lives, such as shift workers, as well as corporate consultants and accountants who had to travel a lot for their work.

There was a wish expressed to get into the self-management group. Exactly, because in my case mechanics say ‘Then I’d rather do my exercises at home and then I don’t need to … because I’m away. I couldn’t do it.’ That also exists. It’s rare, but it exists.

(CM6, FG1)

Personal reasons reported by case managers as reasons for a possible mismatch involved time-dependent variables, such as not being in any pain at the moment, plans for vacation during the 13-week training period and issues such as a deceased family member. Participants who were reported to need a workplace change (some Module C-cases), were seen as not being a good match for any of the groups.

While some case managers reported that they perceived the two groups as equally preferable options, many explained that they perceived the self-management group as inferior to the case management group. Perceptions were generally dependent on the module the participants fitted into. While Module B-cases were perceived as receiving comparable care when they were allocated in either of the groups, being allocated into the self-management group in Module A was perceived as being too little or not enough.

I think that it’s simply because they need help now, and they want help now, and I promised something, we will do something, and this Thera-band is simply too little. It is really incredible. Well, there is something—they want something, that’s why they came and then they basically get nothing.

(CM20, FG3)

Not perceiving group equipoise was often reflected in the use of language by case managers, such as talking about “the wrong group” or “nothing”, when talking about the self-management group.

When they’re getting into the wrong group … unfortunately it happened. Stupid.

(CM5, FG1)

Other expressions used by case managers to describe the study design and group allocation were associated with sales pitches.

You try to have the conversation end well. […] I’ll try to sell it more neutrally now.

(CM20, FG3)

Finally, the language used by case managers to describe group allocation resembled a lottery, with winners and losers.

Well, it is a 50/50 chance.

(CM5, FG1)

#### 3.1.3. How Does It Make Them Feel?

##### Emotional Reaction

Several case managers reported that they were tensed to receive the results of the randomization procedure and that they felt relieved when participants were allocated to the case management group. This gave them a sense of accomplishment and compensation for their efforts.

Yes, there needs to be a sense of achievement at some point, when you bend over backwards like that.

(CM5, FG1)

Several case managers reported feeling disappointed or angry when a participant was not allocated to the case management group.

With the first two, three, I was pretty disappointed, because they all got into the self-management group. So, everyone immediately got into the wrong group.

(CM5, FG1)

Some case managers reported feelings of guilt when participants were allocated to the self-management group and a sense of defeat when this happened repeatedly. Few case managers said that they did not care about the results of the randomization procedure or that they had learned to cope with the allocation of participants to the self-management group.

Gosh, it doesn’t really bother me to be honest. It’s not my fault.

(CM16, FG3)

#### 3.1.4. What Is the Perceived Impact for the (Insurance) Company?

##### Impact Reputation

Case managers reported that they were hoping for an improved image and a better bond with the companies through the project. Some case managers reported that there had been participants who were included in the trial who told colleagues about their positive experiences with the project.

We now also have three that are training, but luckily everything is very positive, and that news spreads, and they really had good experiences. Two are already finished, have completed it, and really say that their pain decreased in the shoulder and knee and they are really satisfied. Luckily, this news also spreads […]

(CM20, FG3)

These kinds of positive examples were also used by some of the case managers as advocates who would report their experiences in the company magazines. Other case managers reported negative news being spread in the companies following allocation to the self-management group, which was perceived as having a negative impact on recruitment.

It was a bit tough to start with, because my first three got into the self-management group and the rumors spread: ‘You don’t need to go there, you’ll only get into the self-management group.’

(CM15, FG3)

Many of the case managers expressed a fear that negative rumors would be spread in the companies and, in extreme cases, that this could harm the insurance company’s image.

Because we asked ourselves not long ago, whether we would do such a project again and at the moment, we are actually really thinking that the risk of damaging the image is higher than I can say is a positive thing, at this moment. But that’s a very subjective estimation at the moment. We have to wait and see if it turns out to be effective. But it’s simply, definitely associated with quite a risk, which can be destroyed in such a factory.

(CM14, FG3)

### 3.2. Effect on Participants

#### 3.2.1. How Do They Perceive the Randomization and Allocation into Groups?

##### Comprehension (Necessity Study)

Case managers reported that it was difficult for many potential participants to understand the study design, as well as the randomization method.

Well, I also have this problem that I often really have the feeling, they simply don’t understand this study design. […] I regularly get people from the self-management group, who regularly call me and ask me, ‘What else can I do, when can I start the training?’ I’ve got a schedule, where it’s actually written out pretty clear that it starts after one year, but understanding the system a little bit, is really, really hard for a lot of them.

(CM7, FG3)

Case managers also reported that they perceive participants as not caring about the study itself, but that they were hoping to get better treatment by participating in the study.

##### Group Perception/Expectations

Most case managers reported that participants would enter the conversation with strong preferences for a group. Usually, this was said to be the case management group.

In my case, everybody wants to get into the case management group. I didn’t have a single person who said: ‘Gosh, a little bit of exercising at home.’ … No.

(CM5, FG1)

However, in some cases, participants were said to have a preference for the self-management group.

And I, by the way, heard from a worker that told me ‘Case management twelve times or two times a week over twelve weeks, I can’t do it. I could do a bit at home.’ So, this type also exists, who says: ‘No, I wouldn’t go anywhere. I would do something at home.’

(CM4, FG1)

In Module B, it was said to not matter much, because participants were expecting to be able to be going into rehab either way. However, for Module A, participants were said to perceive being allocated into the self-management group as being unlucky and receiving nothing in return for participating in the study.

I already heard: ‘I mean, if I’m unlucky, I’ll get nothing from you.’

(CM12, FG2)

Several case managers reported that participants who were not previously informed by an occupational physician or had read about the study on a poster, would be most neutral about the project, and did not enter the conversation with expectations.

What got better, I’m going back in time a bit, is that ever since the letters were out and it didn’t go through the occupational physicians anymore, they (participants) call us directly and we can give them the first/initial information. That means that they don’t come to the appointment with the wrong expectations. I find that helpful.

(CM8, FG2)

Some case managers expressed a negative prognosis for the future, by anticipating that the more people who are recruited in the case management group, the higher the expectations of possible future participants will be.

[…] I think that the more who are going to participate, the higher the expectations: ‘I want to go there (training center), so why do I get the Thera-band?’ I think it will get worse. I don’t think it will get better.

(CM20, FG3)

#### 3.2.2. How Do They React?

##### (Emotional) Reactions

Some case managers reported that participants reacted positively to allocation into the self-management group, when they understood the necessity of both groups for the study.

In our case, or in my case, when I lead the conversations, I couldn’t say that they are that disappointed. Because I try to point out from the start that we need both groups to get the results in the end. So that’s why I couldn’t really say that there’s a lot of disappointment. Of course, with Module A, it isn’t the nicest option that you can only start the training after one year, but generally, they receive the news positively, in my experience.

(CM18, FG3)

The majority of case managers reported the disappointment of participants, mostly as a reaction to not being allocated to their preferred group.

[…] Giant disappointment. That someone dropped out immediately. Immediately said, you can throw away that piece of paper, he’s dropping out. Such a stupid Thera-band, he’s got that at home himself and he also has an exercise machine at home that he never sits on. […] They are utterly disappointed. I’ve now got three in the self-management group, as I said, one of them dropped out, the second one I couldn’t get a hold of, I haven’t heard anything. He also went out with the Thera-band and was utterly disappointed.

(CM20, FG3)

Few participants were said to react with anger.

[…] He slammed the door in the end and said: ‘This is bullshit that I get into the self-management group, I need the work-related rehab. Everything else is bullshit for me.’ And he really got louder, and I was really, really glad when he was gone. He also didn’t fill in the questionnaire. But I knew at the beginning that he wouldn’t do that. But he really got angry and … yes.

(CM7, FG3)

Some case managers said that they saw no reaction from the participants, but that they were sometimes unreachable after recruitment and were not expected to fill in any questionnaires or do any training, irrespective of group allocation.

Yes, I guess, those are the ones you don’t get anything back from. Or also the administration I guess, if I’m not in the project (group) that I want, I think that partly they don’t send anything back. Some are just sitting it out, and also in the case management group. (Person Y, project leader) also said we should ask them about it. But I can write to them, I can call them, they won’t pick up the phone anymore. […]

(CM2, FG1)

##### Impact on the Study

Some case managers reported that they had experienced participants dropping out of the study as a reaction to allocation to a non-preferred group.

In my case, he really got into the wrong group. He really wanted to get into the case management group, Module A and got into the self-management group. I did get angry. Okay. Then he says: ‘Yes, okay, well.’ Then I said: ‘You can, as my colleague told you, get into the case management training after twelve months, that’s no problem.’ ‘Yeah, okay.’ Two days later, three days later, I got a call: ‘Ah, (name case manager). Ah, somehow, no, I’d rather …’ so I said: ‘I can’t force you to do it. If you don’t want to do it …’

(CM3, FG1)

This reaction was already anticipated by some case managers because some participants would ask for reassurance that they could drop out of the study at any time before being randomized.

## 4. Discussion

The aim of this study was to explore factors affecting the recruitment of employees with MSDs to a multicenter work-site RCT from the perspective of recruiting case managers. Focus group discussions were used since previous studies had demonstrated how qualitative methods can help to identify and overcome barriers of recruitment to RCTs [8,10]. Despite efforts to train the case managers in becoming acquainted with the study design before the start of the trial and supportive material during the trial (i.e., field manual, best practice information), randomization was identified as a major challenge for recruitment.

In line with previous studies [14,15,16,17,18,19], case managers in our study reported that communicating about the randomization design was effortful and time-consuming. They reported that they understood the necessity of the randomization design for the study, however, the concept of randomization itself might not have been clear to all case managers, as some reported knowing beforehand which group a participant would be allocated to. A systematic review found that a key barrier for recruiters was a poor understanding of research and difficulties communicating about trial methods [10]. Several previous studies had found that recruiters have treatment preferences and that a perceived lack of equipoise between RCT treatment options can make recruitment challenging [4,14,20,21,22]. Individual equipoise has been described as the uncertainty of an individual about the superiority of a treatment [23]. As had been documented in other studies [22], the case managers in our study expressed different levels of uncertainty, ranging from ambivalence to a clear preference for one of the groups, which caused them considerable discomfort. When it comes to the group allocation in Modules A and C especially, several case managers reported that they perceived the control arm of the study as inferior to the intervention arm. This was either reported to be the case because they perceived the control arm as a poor alternative to the intervention arm, or because they felt that there was a mismatch between participants and the group to which they were allocated. Reported reasons for a group mismatch included a mismatch in the group preference of participants, their age or gender, their profession, and personal reasons that made either group difficult to attend. Group perceptions were further highlighted by the language case managers used to describe randomization allocation. As had been found in other studies, communicating the research design to participants was described as a sales pitch, or like a lottery, with winners and losers [10]. The communication style of recruiters was found to play an important role in participant understanding of RCTs and their willingness to join such a study [8,10]. Perceptions of group mismatch were reported to be associated with guilt, anger, and disappointment. Blinding case managers and/or participants to the conditions, although impossible in the current study design, would have potentially taken away many of the challenges found here.

In order to cope with the cognitive and emotional difficulties resulting from the perceived lack of equipoise and the challenges of communicating the study design to participants, case managers employed different strategies. Next to emphasizing the individual’s situation in the first meeting instead of emphasizing the trial, case managers put effort into providing participants with alternative measures to the standard care available to them. This was specifically allowed by the study design but seemed to be perceived as a sort of compensation for allocation into the control arm of the study. Arguably, this effort was more than employees would usually receive outside of the study. Some case managers questioned whether this would contaminate the study results, as would be the case if case managers implemented alternatives not usually available to employees in standard care [24]. We cannot control for this, however, since the trial includes different health insurance funds with different standard care offers available for their respective employees. Another strategy employed by some case managers was to not offer participation in the trial in order to prevent people from being allocated to the control arm of the study. Similarly, other studies have found that recruiters can act as gatekeepers, who only approach participants they deem suitable for participation [10]. Consequently, employees who would actually be eligible for participation in the study might not be approached in the first place, further affecting recruitment numbers.

As had been found before, case managers generally reported being happy to be involved in the study, because it was seen as beneficial to be able to provide participants with novel treatments [10,15,16]. Being able to offer participation in novel treatments was found to encourage confidence and loyalty in participants [10]. Case managers in our study reported that they felt this project might improve the image of the health insurance funds and strengthen the bond with the companies, however, this, in turn, meant that not being able to offer participation in the intervention arm of the study, caused a conflict for case managers. Fletcher and colleagues [9] found that recruiters were concerned that offering participation in a study might affect participants’ trust negatively and that it was perceived as emotionally detrimental to offer participation to ill patients, in case they were placed in the control group. We found something similar, in that case managers found it especially difficult to offer participation to employees who were at risk of losing their job because of their disorders (Module C-cases), although this only concerned a very few participants. This might be more likely in cases where case managers had known employees personally. Negative news being spread about the study in companies was also perceived as bad for recruitment, as well as for the image of the health insurance funds and companies. Case managers might fear that employees would be lost as customers.

Studies show that recruiters can perceive barriers to recruitment as more closely related to patients than themselves, as they are perceived to have a poor understanding of studies and a low motivation to participate in research [9]. Similarly, case managers in our study reported that some participants had difficulties understanding the study design. Participants were also said to often have prior expectations when entering the conversation with the case manager (e.g., due to previous conversations with colleagues/employers/occupational physicians) and would react with anger and disappointment when being allocated to a non-preferred group. As a consequence, case managers felt that they had to put effort into managing expectations and needed to explain that they had no influence on the outcomes of the randomization procedure, and thereby which treatment group a participant would be allocated to. Llewellyn and colleagues found that the majority of participants who refused to take part in an RCT reported an aversion to randomization as their primary reason [25], and that decisions about group allocation that are based on chance were previously described as a barrier to participation [14]. These emotional reactions might be explained by learned helplessness or pain self-efficacy [26,27]. However, we can only speculate about this, since we could not include participants’ perspective in this study. Instead, we have to rely on the interpretations of the case managers. Of course, this also raises concerns about the overall internal validity of the RCT at hand. Reviews and case studies have previously been conducted to explore whether preference effects may lead to inaccurate conclusions about treatment efficacy [9,28]. While King and colleagues [9] did not find support for this hypothesis, Janevic and colleagues [28] suggested that there might be an effect on the motivation and adherence of participants, which might bias efficacy evaluations. Several case managers in our study reported that they had experienced participants dropping out of the study as a consequence of not being satisfied with the randomization result and being allocated to a non-preferred group.

A strength of using focus group discussions is that they allow for exchanges between their participants. They can thereby simultaneously act as an exploratory method and as a strategy to stimulate the exchange of best practices and materials used. Accordingly, information gained from the discussions was used for the adaption of best practice information sheets and newsletters distributed among all case managers. In regular meetings with the case managers, the research team encouraged the exchange of recruitment strategies and provided individual feedback to case managers on specific questions. Elliott and colleagues also recommend support to enable recruiters to feel more comfortable in discussing participant uncertainty and explaining to participants that they are eligible for both groups, while explaining the rationale behind randomization [8]. Extensive communication training, possibly including role play, might help recruiters to successfully convey the study design and to make them aware of how language can shape the expectations and perceptions of participants. The framing of the intervention could have led to nocebo effects, meaning that there might not be a beneficial effect of self-management because participants did not expect an effect [28,29]. Since case managers reported finding it difficult to deal with the prior expectations of possible participants, which they, for instance, had received from conversations with occupational physicians or their employer, it could be beneficial to restrict recruitment activities to case managers. Alternatively, it might be necessary that all project members are better trained in conveying the study design when talking to possible participants. After all, in case of implementation of the program into standard care, collaboration between project partners is vital for optimal program use. Alongside its strengths, there are a number of limitations to this study. Firstly, we were not able to include the perspective of eligible trial participants in our study, which might have given us more insight into perceptions surrounding the study design and employees’ real reasons for participating or not participating in the study. We do collect information from participants and nonparticipants through questionnaires, however, as part of the trial evaluation, which we can review simultaneously. Secondly, focus groups can have the disadvantage that individual participants might not feel comfortable expressing critical thoughts, however, the case managers in this study seemed eager to exchange experiences, and the challenges of recruitment seemed to be openly discussed.

## 5. Conclusions

This study confirmed that the challenges of RCT designs for the recruitment of participants known from clinical settings also apply to complex multicenter worksite interventions. The case managers participating in our study all had very different initial situations, as well as different recruitment goals and recruitment numbers when the focus group discussions were conducted. Nevertheless, randomization and perceptions surrounding the study design with resulting effects on recruitment seemed to be similar across case managers. Despite efforts to contain issues related to the study design before the start of the trial, randomization was identified as an important challenge for case managers when recruiting employees.

We were able to gain insight into the effect of randomization on the recruitment efforts of case managers, which assisted the development of strategies to address challenges as the trial proceeded. Regular meetings and follow-up focus group discussions with case managers one year later will provide information on the effectiveness of the targeted strategies. Nevertheless, as has been previously suggested, there is still a need to develop effective interventions aimed at those recruiting to trials.
